# Inspired by the human placenta: a novel 3D bioprinted membrane system to create barrier models

**DOI:** 10.1038/s41598-020-72559-6

**Published:** 2020-09-24

**Authors:** Anna-Elisabeth Kreuder, Aramis Bolaños-Rosales, Christopher Palmer, Alexander Thomas, Michel-Andreas Geiger, Tobias Lam, Anna-Klara Amler, Udo R. Markert, Roland Lauster, Lutz Kloke

**Affiliations:** 1 Medical Biotechnology, Technical University of Berlin, Berlin, 13355 Germany; 2 Cellbricks GmbH, Berlin, 13355 Germany; 3 Placenta Lab, Department of Obstetrics, University Hospital Jena, 07747 Jena, Germany

**Keywords:** Tissue engineering, Cancer models, Gastrointestinal models, Skin models, Urogenital models

## Abstract

Barrier organ models need a scaffold structure to create a two compartment culture. Technical filter membranes used most often as scaffolds may impact cell behaviour and present a barrier themselves, ultimately limiting transferability of test results. In this work we present an alternative for technical filter membrane systems: a 3D bioprinted biological membrane in 24 well format. The biological membrane, based on extracellular matrix (ECM), is highly permeable and presents a natural 3D environment for cell culture. Inspired by the human placenta we established a coculture of a trophoblast-derived cell line (BeWo b30), together with primary placental fibroblasts within the biological membrane (simulating villous stroma) and primary human placental endothelial cells—representing three cellular components of the human placental villus. All cell types maintained their cell type specific marker expression after two weeks of coculture on the biological membrane. In permeability assays the trophoblast layer developed a barrier on the biological membrane, which was even more pronounced when cocultured with fibroblasts. In this work we present a filter membrane free scaffold, we characterize its properties and assess its suitability for cell culture and barrier models. Further we show a novel placenta inspired model in a complex bioprinted coculture. In the absence of an artificial filter membrane, we demonstrate barrier architecture and functionality.

## Introduction

Barrier tissues of the human body present the interface to the outside world, for example the skin, lung and gut. Last but not least the placenta represents the interface between maternal and fetal blood circulation and thus a secondary barrier, e.g. for xenobiotics and pathogens, which need to first enter into the maternal blood before they may pass into the fetal circulation. Other barrier tissues separate compartments within the human body, as the blood–brain-barrier or kidneys do. Barrier organs regulate homeostasis and protect organs from harmful influences. In vitro barrier models are useful to estimate if a substance can reach a certain compartment to exhibit (wanted or unwanted) effects or to study the influence on barrier integrity itself.

Barrier models are most often established on a scaffold that creates two compartments in a tissue culture plate: the volume in the culture well and the internal volume of the insert. Commonly used scaffolds are technical membranes, also referred to as filter inserts, made of PET (polyethylene terephthalate), PC (polycarbonate), or PTFE (polytetrafluoroethylene) with defined pore sizes. The properties of these scaffolds must be considered, since the material, pore size and porosity may interact with a tested molecule^1,2^ and can impact cell behaviour^3,4^, thus presenting a barrier themselves. Although coatings with ECM components are commonly used to improve cell attachment and survival^5–7^, few attempts have been successful to fully replace filter membranes^8–10^.

In this work we present a biological membrane system as an alternative for filter membranes: the Membrick contains a membrane based on gelatine instead of a technical membrane, and can be used in 24 well culture plates. With this biological membrane we are addressing the potential influence of artificial material on cells or barrier function and provide a 3D cell culture environment. We characterized and compared the Membrick to widely used PET filter membrane systems and showed its potential for epithelial and endothelial cell culture. Moreover we present the possibility to print cells into the biological membrane and show a coculture system inspired by the human placental barrier.

Pregnancy poses a particular challenge to substance safety assessment, since pregnant women are excluded from clinical trials due to potential health risks. Available model systems have certain limitations: mice are widely used but do not resemble human placentation in many aspects since the placenta is one of the most species-specific organs^11,12^. The most complex and accurate testing system available today is placenta perfusion, which is useful to predict placental drug transfer at term. Placenta perfusion experiments are limited in the maximal perfusion duration of 12h, high donor to donor variability, low throughput, and work intense set-ups^13,14^. On the other hand, in vitro models are easier to handle, more accessible and have a higher throughput, but lack complexity and therefore biological relevance. Multiple biophysical and biochemical factors in the microphysiological cell environment impact tissue barrier functionality, for example blood flow, matrix rigidity and interaction of different cell types^15–18^. Recently, advances in placental models have been achieved using primary villous trophoblast cells^5^ or coculture of trophoblast cells with microvascular endothelial cell line HPEC-A2^6^, or HUVEC and dermal fibroblasts (NHDF)^19^. In addition, placental models have also been introduced into microfluidics, mimicking blood flow at the placental barrier^10,20–23^.

**Figure 1:**
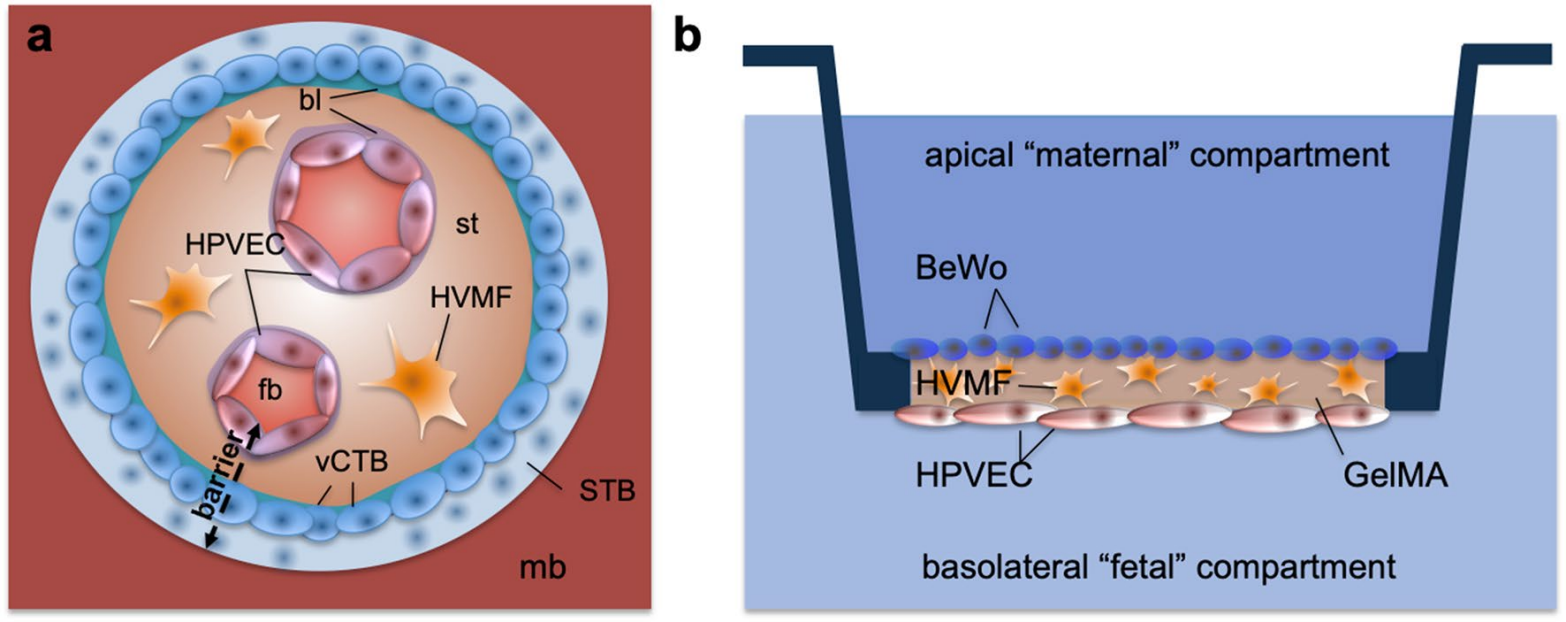
Scheme of placental barrier (gestation week 5), and its model. A placental villus (**a**) contains fetal blood vessels (fb), which are lined with endothelial cells (HPVEC) and surrounded by a basal lamina (bl). Mesenchymal stroma (st) with fibroblasts (HVMF) surround the vessels. The outermost layer of the villus is built out of villous cytotrophoblast cells (vCTB), with a basal lamina (bl), which fuse into one syncytiotrophoblast (STB) that is in direct contact with maternal blood (mb) in the intervillous space. At this stage of development the placental barrier consists of endothelial cells (with basement membrane), stromal tissue, cytotrophoblast cells (with basement membrane) and syncytiotrophoblast. Analogous the scheme of the placental barrier model on the Membrick, (**b**) the biological membrane is based on methacrylated gelatine (GelMA), containing human villous mesenchymal fibroblasts (HVMF). Human placental vascular endothelial cells (HPVEC) are cultured on the basolateral side, while the trophoblast cell model BeWo is cultured on the apical side of the biological membrane.

Our goal was to establish a coculture that rather reflects the natural cell environment and architecture of the early placental barrier, see Fig. 1. The placenta starts developing with the implantation of the blastocyst into the uterine wall approximately at day 5 post conception (p.c.). At this stage, the first linage separation took place and separated the inner cell mass, or embryoblast and the outer cell layer, the trophoblast (trophectoderm). A portion of the trophoblast fuses to form the syncytiotrophoblast, which shows an invasive phenotype at this point of pregnancy, and is in charge of the implantation as it digs the embryo into the endometrium. Trophoblast cells underlying the syncytiotrophoblast stay proliferative and expand the syncytium as they fuse into it, those (mononucleated) cells are called the villous cytotrophoblast. The syncytiotrophoblast further forms vacuoles starting at day 8 p.c., which eventually become the blood filled intervillous space, as trophoblast cells open up maternal blood vessels in the end of the first trimester. The syncytiotrophoblast structures, which grow into the endometrium like branches since the implantation, contain villous cytotrophoblast cells around day 8 p.c. and are called primary villi. Around day 14 p.c. mesenchymal cells grow into the villi, which now represent secondary villi. Starting day 18–20 p.c. vasculogenesis starts within the villous mesenchyme, characterizing the tertiary villi. In the end of the first trimester blood vessels of the placenta connect to fetal blood vessels and enable substance exchange between mother and fetus. The tissue structures separating the fetal and the maternal blood, the placental barrier, consists of: (1) fetal blood vessels and (2) their basal membrane, (3) mesenchymal stroma with fibroblasts, (4) basal membrane of trophoblast portion with (5) the cytotrophoblast layer and 6. the syncytiotrophoblast, see also 1. From then on to term the stromal layer within the villi thins, the cytotrophoblast cells become fewer; lastly the basal membranes of fetal endothelium and of the trophoblast fuse^24^.

At term, the placental barrier consists of (1) fetal endothelium, (2) the fused basal membrane and (3) the syncytiotrophoblast, and measures overall 3–5 micrometer^25,26^. Inspired by the tertiary villus architecture in early pregnancy, we created a model without a technical filter membrane, that contains three human placental cell types. A biological membrane containing human villous mesenchymal fibroblasts (HVMF) was bioprinted, to resemble the stroma of placental villi. Human placental villous endothelial cells (HPVEC) were seeded on the basolateral (well-facing) side, and BeWo b30 cells on the apical side (in Membrick), as a model for villous trophoblast cells. Barrier formation of monotypic BeWo and coculture with HVMF was characterized in permeability assays using a paracellular pathway marker (Lucifer Yellow, LY) and in daily measurements of transepithelial electrical resistance (TEER) during one week. Additionally, we showed the spatial arrangement of BeWo cells and endothelial cells in coculture with HVMF after two weeks of culture.

## Results

### Biological membrane: technical details

**Figure 2:**
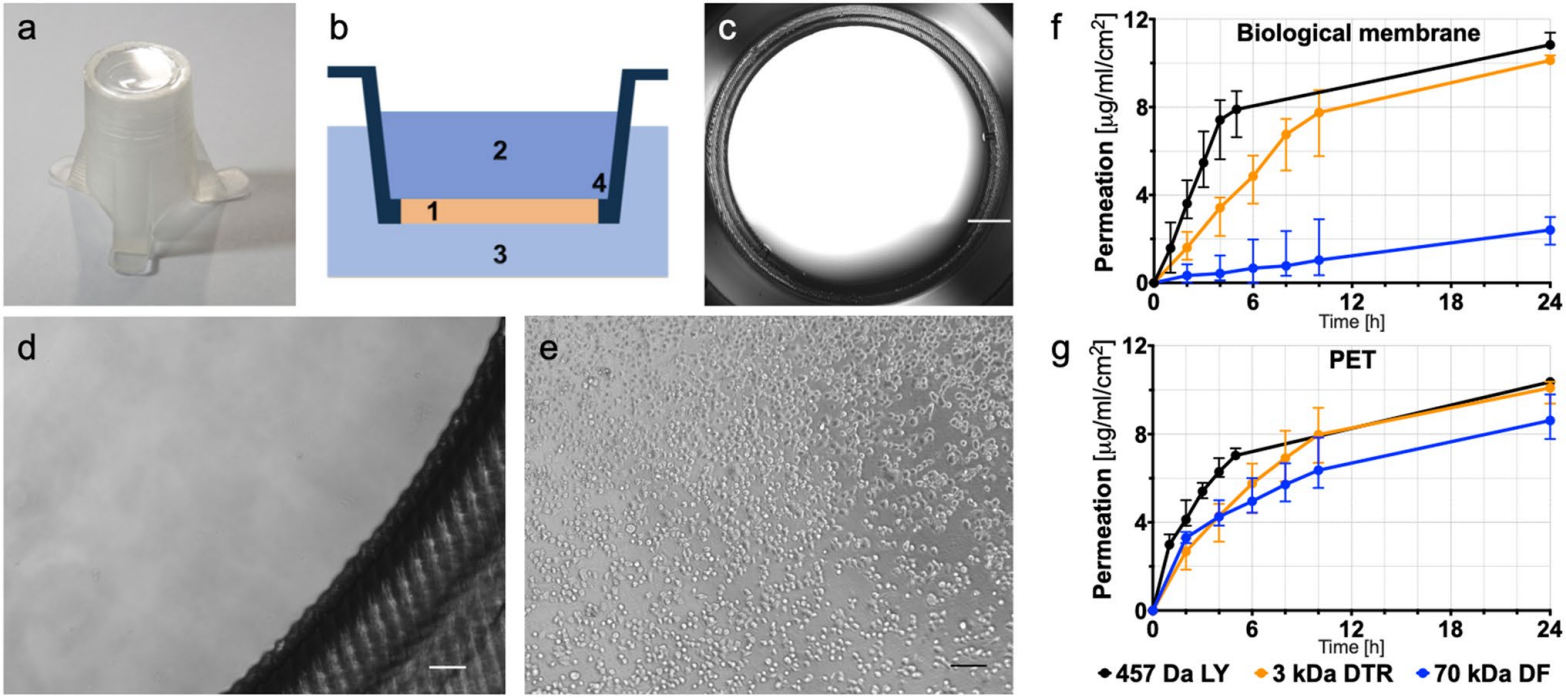
Biological membrane system (Membrick) and its characteristics. The cell culture insert is designed for hanging cultivation in a 24 well plate (**a**). Depiction of cell culture insert scheme (**b**): biological membrane (1) separating two compartments (2, 3). The Membrick body has arms for a hanging configuration in 24 well; the bottom of the cylindrical body includes a pipette rest for facilitated media exchange (4). The biological membrane is transparent in bright-field microscopy (**c**, **d**). Cells seeded on top of the biological membrane can be observed continuously through the membrane (**e**). Scale bars represent $1000\;\upmu$m (**c**), and $100\;\upmu$m (**d**, **e**). Permeability of the biological membrane (**f**) compared to PET (**g**) with $1\;\upmu$m pore size for Dextran-FITC and -Texas Red (70 kDa and 3 kDa) and Lucifer Yellow (457 Da). Dextran permeability for biological membrane ${\text{ n }}=6$, and LY ${\text{ n }}=5$, permeability for PET ${\text{ n }}=7$, and LY ${\text{ n }}=5$. Median and error are displayed.

We developed a cell culture insert with a biological membrane, further referred to as ’Membrick’. The Membrick body features arms for a hanging configuration in standard 24 well plate format, see Fig. 2a,b. The Membrick’s biological membrane area measures 0.28 $\hbox {cm}^{2}$, is based on gelatine methacrylate (GelMA) and transparent, see Fig. 2c,d. Its elasticity described as Young’s modulus is $24 \pm 0.35$ kPa. The thickness of the biological membrane is $363.3 \pm 31.5\;\upmu$m (${\text{ n }}=6$) (see Supplementary Fig. 1 online). Seeding areas on both sides of the biological membrane have a surface of $0.33\;\hbox {cm}^{2}$, corresponding to 96 well format, and include a pipette rest for facilitated medium exchange. Cells can be cultured on both sides of the biological membrane, on the apical side, as well as on the basolateral side. Seeded cells can be observed non-invasively and continuously using any kind of optical microscopy, due to the transparent nature of the biological membrane, see Fig. 2e and Supplementary Fig. 3.

Since gelatine closely resembles the natural cell environment, with both biochemical and biophysical cues, coating with ECM is not necessary for many cell types and handling steps are reduced to a minimum. Additionally, due to its hydrogel character the biological membrane is permeable for solutes. The object of investigation was the extent to which the biological membrane is permeable and how it compares to a common filter membrane. To characterize the permeability of biological membranes we conducted assays with different molecular weight molecules and performed TEER measurements.

### Permeability of biological membrane

To assess solute permeability across the biological membrane, its electrical resistance was measured (basic resistance) over the time course of 2 weeks and compared to a PET membrane in 24 well format with $$1\;\upmu $$m pore diameter. The biological membrane showed a substantially lower resistance with $$18.22 \pm 1.6$$$$\Omega \hbox {cm}^{2}$$ compared to PET membrane with $$36.2 \pm 2.8$$$$\Omega \hbox {cm}^{2}$$ , see Supplementary Fig. 1 online.

Further characterisation of membrane permeability was performed using different weight molecules of 457 Da Lucifer Yellow (LY), 3 kDa Dextran-Texas Red (DTR) and 70 kDa Dextran-FITC (DF) in serum free medium. The permeation of PET and the biological membrane for small and medium sized molecules (457 Da LY and 3 kDa DTR) was similar, while 70 kDa DF had a tendency to permeated slower through PET and visibly slower through the biological membrane see Fig. 2f,g. After 24h $10.83\;\upmu$g/ml/$\hbox {cm}^{2}$ LY and $10.13\;\upmu$g/ml/$\hbox {cm}^{2}$ DTR permeated through the biological membrane, whereas $10.37\;\upmu$g/ml/$\hbox {cm}^{2}$ LY and $10.1\;\upmu$g/ml/$\hbox {cm}^{2}$ DTR permeated through PET with $1\;\upmu$m pores, respectively, see Fig. 2f,g, as well as Supplementary Fig. 2 online. At 4 h the apparent permeability coefficient ($\hbox {P}_{{app}}$) for LY corresponded to $1.52\times 10^{-5}$ cm/s for the biological membrane, and $1.28\times 10^{-5}$ cm/s for PET respectively (${\text{ p }}=0.0025$), see Supplementary Fig. 2 online. Passage of DTR was in a comparable range, with no obvious difference in $\hbox {P}_{{app}}$ between Membrick and control $1.14\times 10^{-5}$ cm/s and $1.12\times 10^{-5}$ cm/s respectively). For the high molecular weight DF, the PET membrane displayed a low hindrance, with a permeation of $8.62\;\upmu$g/ml/$\hbox {cm}^{2}$ after 24 h and a $\hbox {P}_{{app}}$ of $9.64\times 10^{-6}$ cm/s (at 4 h). A substantially lower permeability for 70 kDa DF was observed for the biological membrane, through which only $2.41\;\upmu$g/ml/$\hbox {cm}^{2}$ DF permeated at 24 h, and which displayed a $\hbox {P}_{{app}}$ of $1.42\times 10^{-6}$ cm/s at 4 h, (${\text{ p }}=0.0012$).

### Cell culture on biological membrane

**Figure 3:**
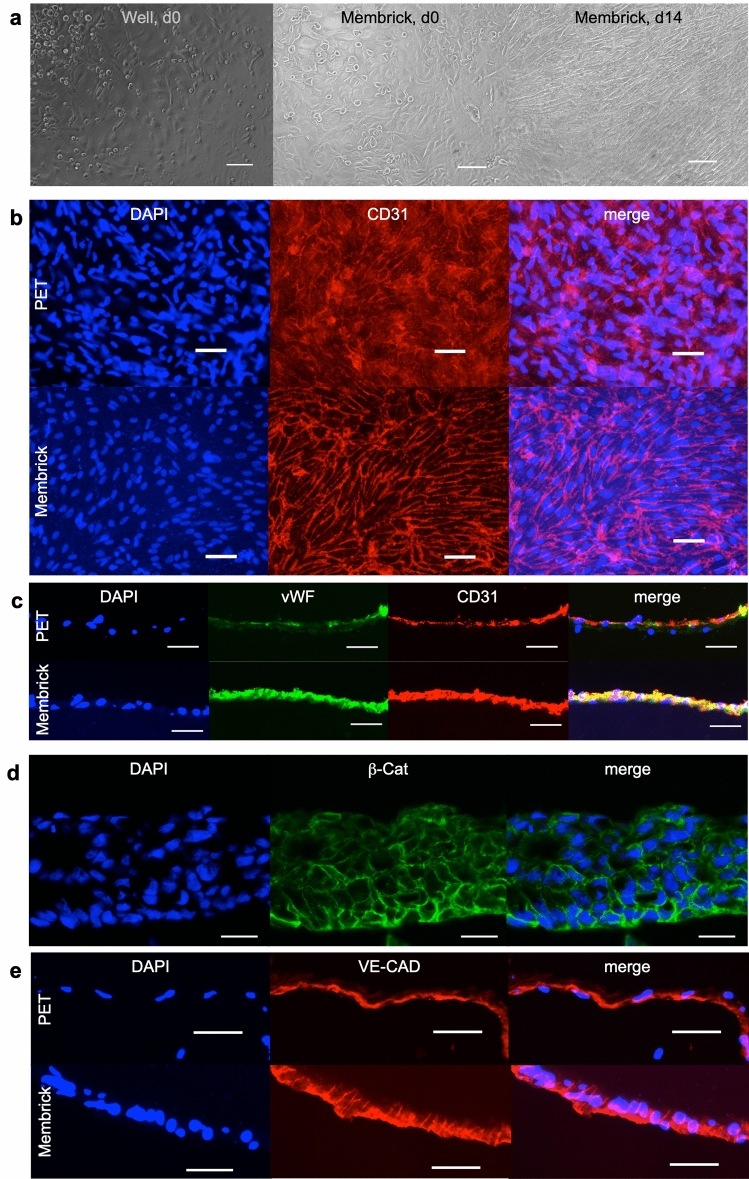
Monotypic culture of placental endothelial cells (HPVEC) and BeWo cells cultured on biological membrane. Cell morphology of HPVEC in bright-field microscopy at the beginning of experiment and after 14 days of culture on the Membrick (**a**), scale bar $100\;\upmu$m. Immunofluorescence staining of HPVEC culture on PET and biological membrane for endothelial specific marker CD 31, on day 12 (**b**). Cross sections (**c**) on day 14, stained for CD31 and von Willebrand factor (vWF), scale bar $50\;\upmu$m. BeWo b30 cells stained for beta-catenin ($\beta$-cat) at day 5 of culture on Membrick (**d**), scale bar $25\;\upmu$m. Comparison of vascular endothelial cadherin (VE-Cad) localization in HPVEC cultured on PET and Membrick (**e**), scale bar $50\;\upmu$m.

As a use case of the Membrick system, we established a coculture with different placental cells to mimic the human placental barrier. We cocultured the commonly used trophoblast-derived choriocarcinoma cell line BeWo, clone b30 either with or without primary fibroblasts (HVMF) and endothelial cells (HPVEC) of placental origin and investigated barrier formation on the Membrick. We chose the choriocarcinoma cell line BeWo b30 as a trophoblast cell model for its ultrastructural similarities to trophoblast cells, in particular for its ability to grow confluent and for its syncytialization abilities^7,27^. Further, BeWo cells show polarized cellular structures and secretion of typical hormones (e.g. hCG, placental alkaline phosphatase, estrogen)^27^ and have transport protein expression similar to primary cytotrophoblast cells (including ion^28^, amino acid^29^, glucose^30^ and immunoglobulin G transporter^31^). Apparent differences between BeWo and primary trophoblast cells are found in low spontaneous differentiation of the choriocarcinoma cells into a syncytium^32^ and in protein expression patterns characterizing their immortal and invasive character^33^. Despite differences, an in vitro model using human choriocarcinoma cells compares favourably to other testing systems, especially to animal models, where placentation is different from human on levels of structure, anatomy, cell types and molecular biology^12,34^. BeWo b30 cells were onto the biological membrane at $10^6$ cells/$\hbox {cm}^2$, in accordance with a previous publication^5^. The cells quickly covered up the entire surface on the Membrick, and appeared to be confluent on day 1, see Supplementary Figs. 4 and 5 online. Beta-catenin ($\beta$-Cat) staining for cell-cell adhesions appeared positive in fluorescence stainings (day 5), see Fig. 3d, and BeWo cells showed multi-layered growth, which is a known unphysiological feature of the BeWo cell line^7^.

To investigate how endothelial cells grow on the biological membrane, we seeded HPVEC onto the basolateral side, at a density of $10^6$ cells/$\hbox {cm}^2$. The cells showed a rounded to cobblestone morphology 2 h after seeding, and elongated visibly during 14 days of culture, see Fig. 3a. The growth pattern was emphasized in CD31 stainings, which is a intercellular junction protein (also called PECAM-1) see Fig. 3b. Although HPVEC showed an elongated phenotype in bright-field microscopy on the PET membrane (data not shown), CD31 staining seemed less organized (on day 12). HPVEC cultures were cross sectioned and stained for CD31 and von Willebrand factor (vWF), which is commonly produced by endothelial cells. Here, the signal for both markers appeared more restricted in cells cultured on PET and discontinuous, see Fig. 3c. Further staining for the endothelial specific adherens junction marker vascular endothelial cadherin (VE-Cad) was present in both, cultures on the biological membrane as well as on PET, and indicated confluent cell growth, see Fig. 3e.

### Cell integration into the biological membrane

**Figure 4:**
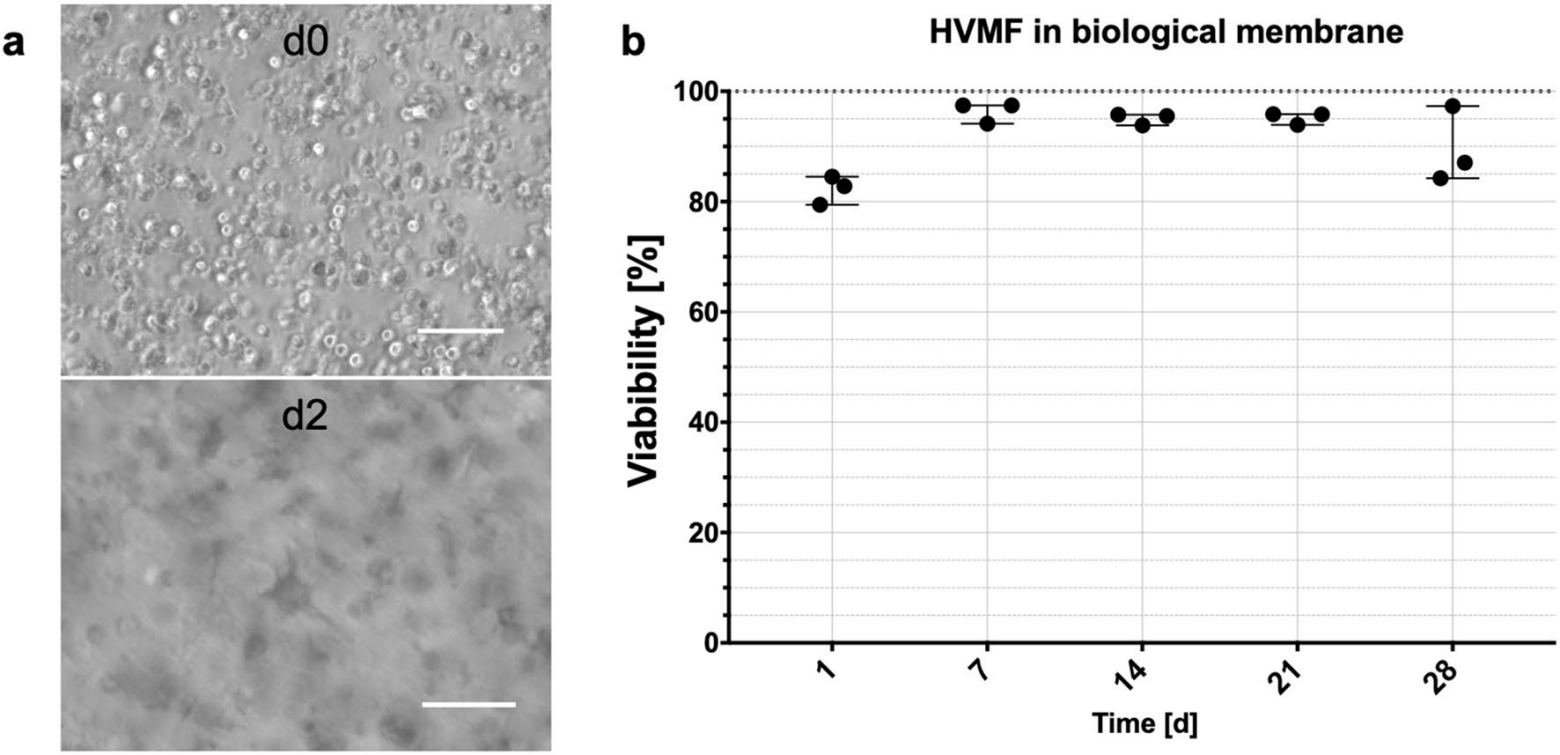
Bioprinted biological membrane with primary human fibroblasts HVMF. Light microscopic image of cells right after printing process and after 3 days in culture (**a**), scale bars $100\;\upmu$m. Cell viability of HVMF over four weeks of culture (**b**), for each time point individual experiments (${\text {n}}=3$) with range are displayed.

Translating the placental barrier components towards an *in vitro* model, placental fibroblasts were bioprinted into the biological membrane, before endothelial and/or BeWo cells were seeded onto it. Since BeWo and HVMF can be cultured in various media, endothelial cell medium MV2c was chosen as culture medium for single and coculture of all cell types. First, the survival of cells bioprinted into the biological membrane was investigated throughout four weeks. Cell spreading occurred within the gel and on the surface within the first three days after printing, see Fig. 4a. Viability throughout the experiment was high with 82.3% ($\pm 2.6$) at day 1 and for the next three weeks above 95%. After 4 weeks the viability was 89.5% ($\pm 6.9$) at day 28, see Fig. 4b.

### Coculture of placental cells on Membrick

**Figure 5:**
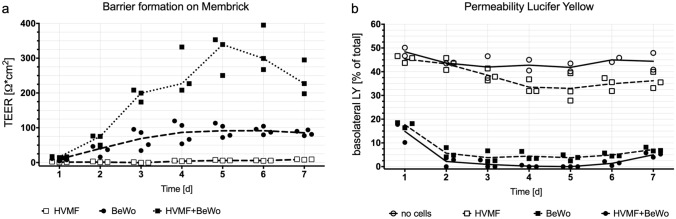
Cell (co)culture on Membrick. Primary placental fibroblasts (HVMF) were included into the biological membrane and viability was investigated throughout four weeks (${\text {n}}=3$ for each time point), **a**; average and range are displayed. Barrier formation of monotypic culture (BeWo on or HVMF in biological membrane) or coculture of both represented by TEER measurements (**b**, ${\text {n}}=3$) and permeability assay with Lucifer Yellow (**c**, ${\text {n}}=3$, empty control ${\text {n}}=2$). Median and individual samples are displayed.

For cocultures biological membranes with fibroblasts were cultured for 3  days, before BeWo cells and/or endothelial cells were seeded onto them. Membricks were cultured for 12 days after the BeWo (and HPVEC) cells were added, so for a total of 14 days. Pictures of each individual sample were taken each day using bright-field microscopy to follow cellular growth and membrane coverage. BeWo cells grew on the cell-free biological membrane and on the biological membrane with fibroblasts included (see Supplement Fig. 3), and appeared confluent starting day 1 of coculture until day 12. Cross sections of cocultures indicated no decrease in biological membrane dimensions, e.g. as a consequence of cell culture (see Supplementary Fig. 6 online). Transepithelial electrical resistance was measured for 7 days and increased in single BeWo culture on the Membrick, reaching a maximum median resistance of $91.5\;\Omega \hbox {cm}^{2}$ at day 6. Coculture (BeWo/HVMF) reached $339.3\;{\Omega }$cm^2^ at day 5, see Fig. 5a. Paracellular permeation of low molecular weight LY decreased in both single cultures and cocultures, and dropped below 8% and 4%, respectively at day 2 of culture, remaining below those levels for the following 4 days, see Fig. 5b.

**Figure 6:**
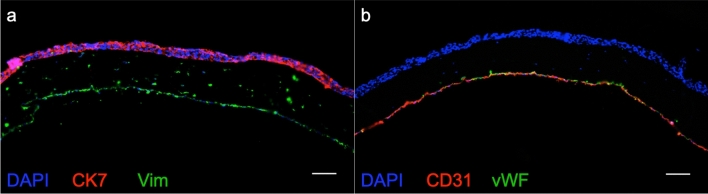
Immunofluorescence staining of coculture on biological membrane at day 12: HVMF printed into the biological membrane, cocultured with HPVEC on basolateral side and BeWo cells on apical side. Cells within membrane stain positively for vimentin (Vim), while apical cells showed cytokeratin 7 (CK7) staining (**a**). Endothelial marker von Willebrand factor (vWF) and CD31 stain positively for cells on basolateral side of biological membrane (**b**). Scale bars $100\;\upmu$m.

Coculture of all three placental cell types—fibroblasts, BeWo and endothelial cells—on the biological membrane of the Membrick was cultivated for 12 days. Upon immunofluorescence staining on day 12 fibroblasts within the biological membrane and endothelial cells on the basolateral side stained positively for vimentin, whereas apical cells stained positively exclusively for cytokeratin 7, and showed multi-layered growth pattern, see Fig. 6a. HPVEC cultivated on biological membrane showed monolayer growth and stained positive for CD31 as well as for vWF, see Fig. 6b.

## Discussion

Barrier models represent human organs like lung, gut, kidney, blood-brain-barrier, skin or placenta. These models are used to study barrier (patho)physiology, response to toxins or to test translocation of substances. Up to now, the basis of most two-compartment barrier models have been filter inserts with pores, which introduce an artificial component, impacting cell behaviour and barrier functionality. Cell function is strongly dependent on the cell micro-environment, substrate stiffness and chemical cues^15,35^.

The objective of this work was to evaluate the potential of a bioprinted, filter membrane-free system for its suitability as a natural environment for barrier models in research and substance testing. The 3D bioprinted biological membrane system we present is based on the gelatine derivate GelMA. Gelatine is a commonly used ECM component in cell culture and its derivative GelMA is well characterized and widely used in 3D bioprinting, which Yue et al. reviewed comprehensively^36^. Many cell types of different tissue origin have been cultured successfully on GelMA: endothelial (HUVEC^37,38^, ECFC^39^) and epithelial cells (Ishikawa^40^, BeWo^41^) as well as cells of mesenchymal origin (NIH-3T3^42^, MG63, osteoblasts^43^).

The biological membrane is thicker than filter membranes, nonetheless it shows comparable permeability for small and medium sized molecules to PET filter membranes (with $1\;\upmu$m pore diameter) and about 50% lower electrical resistance. This difference in resistance indicates higher solute permeability through the biological membrane, and underlines its biomimetic, ECM like character, high water content and a permissive character^44,45^. These features may have an impact on cultured cells, e.g. on the exposure of basolateral membrane transporters to ions, molecules, particles or para- and juxtacrine cell-cell signaling.

In permeability assays no major differences between PET filter and biological membranes were detected towards small and medium sized molecules up to 3 kDa. Larger molecules (70 kDa) on the contrary, traversed notably less (about 50% less within 24h) through the biological membrane than through (non-coated) PET filter membranes ($1\upmu \hbox {m}$ pore diameter). Yet, filter membranes are commonly coated with ECM before utilization, which is discussed to alter permeability. Transwell ($0.4\;\upmu$m pore diameter) membranes coated with Matrigel for example, exhibit transfer rates for FD in the range of $10^{-7}$ cm/s^5^, which is an order of magnitude lower compared to the permeability of the biological membrane. The biological membrane in contrast, since it is based on ECM, does not need additional coating for culture of various cell types and is ready to use. Further, a cut-off towards large molecules as the biological membrane presents, may reflect physiological characteristics of the placental stromal compartment or even the basal membrane. This is in accordance with the placental barrier cut-off of 500–1000 Da at term^46,47^, for which endothelial tight junctions were identified to be responsible for^48,49^. Although the role of the stromal compartment in the placental barrier remains to be elucidated in this context, it could be integrated into current models and investigated using the biological membrane.

As a proof of concept we established a novel filter membrane-free barrier model, inspired by the human placenta. First, monotypic cultures of placental cell types were investigated, before cocultures were established on the biological membrane. The trophoblast cell model BeWo was cultured on biological membranes to investigate barrier formation. The BeWo b30 clone was used before in placental barrier models and was shown to grow confluent, forming a physical barrier^7,50^. For a visually confluent cell layer, we seeded BeWo at $10^6$ cells per $\hbox {cm}^2$, which is also employed for primary trophoblast cells^5^. In other studies, lower cell numbers were used for seeding^6,7^. Although adaptation of cell number may be possible, we did not succeed in growing confluent cell layers on biological membrane over four days using seeding densities between 2.5 and $5.5 \times 10^5$ cells per $\hbox {cm}^2$. The higher seeding density on the biological membrane may be due to the lower substrate elasticity compared to PET, since softer materials do not promote over-proliferation and migration^36,51^. Additionally, the biological membrane’s elastic modulus of 24 kPa reflects the stiffness of placental tissue with 2–22 kPa^52^ more closely than many filter membrane materials like PET with 2–2.7 GPa.

Primary placental endothelial cells grew confluent on biological membranes and showed endothelial specific markers in immunofluorescence stainings. While all markers were likewise detected in cultures on PET membranes, CD31 localization seemed less organized and more restricted than on the biological membrane. This might indicate dedifferentiation of endothelial cells^53^ and points towards the biological membrane as a favourable environment.

To recreate the mesenchymal stromal tissue of placental villi, human placental fibroblasts were integrated into the biological membrane. The survival rate after one day was very high (82%) indicating a gentle manufacturing process. During the following four weeks of culture the viability increased, indicating cell proliferation. After four weeks of culture, viability of fibroblasts decreased to the initial level. This phenomenon might be due to fibroblasts proliferating on the surface and their generally high nutrient consumption, which may restrict nutrient supply to cells inside the gel. Thus, long term cultures of GelMA with integrated fibroblasts is possible, while viability and nutrient restriction need to be considered during the experiment.

Cocultures with fibroblast and BeWo cells was established to investigate the influence of the stromal compartment on barrier functionality. Cocultures reached more than double the TEER value of monotypic BeWo culture. Thus, fibroblasts seem to impact barrier formation. Comparing TEER values to the literature, BeWo culture on the biological membrane reached similar levels (about $100\;\Omega \hbox {cm}^{2}$) as on PET filter membrane coated with collagen or with polymerized GelMA^7,41^. The coculture of BeWo and fibroblasts on the biological membrane system, on the contrary to monotypic BeWo culture, reached more than twice the TEER values ($250\;\Omega \hbox {cm}^{2}$), but were still below levels reported for single BeWo culture on PET membranes (pore sizes of 1–$3\;\upmu$m) coated with Matrigel or collagen IV (about 1200 and 600 $\Omega \hbox {cm}^{2}$ respectively)^5,6^. Differences in TEER values reported in literature are not consistent for BeWo culture, and may be due to different setups, coatings, differences in technical TEER applications, for example placement of electrode, or medium. We noticed for example more consistent results when using an electrode holder we designed ourselves. Throughout two weeks, cocultures presented visually confluent growth, which could be observed due to the biological membrane’s transparent nature, and the system showed structural integrity. Although GelMA, like gelatine, contains restriction sites and is theoretically biodegradable by gelatinases, BeWo cells did not seem to degrade it. In general cytotrophoblast cells are in charge of implantation of the blastocyst into the uterine wall. This invasive phenotype is characteristic for first trimester trophoblast cells only, and comes along with expression of matrix metalloproteinases (MMP)-2 and 9 (also called gelatinase A and B)^54^. Cytotrophoblast cells at later stages of pregnancy do not show the same expression and MMP activity, neither do choriocarcinoma cell lines like BeWo^54^, which are considered a model for the third semester trophoblast. While BeWo cells express MMP-2, and negligible amounts of MMP-9 as well as no tissue inhibitor of matrix metalloproteinase (TIMP-1), they are unable to invade Matrigel^55^. Furthermore, BeWo cells express MMP-11, -14, -15 and -19 on mRNA and MMP-11, proMMP-15 on protein level^56^. The latent and active forms of MMP-2 together only showed moderate gelatinolytic activity^56^. Additionally, functionalization of gelatine attenuates the gel’s degradability, indicating GelMA will be digested slower by MMPs in general^57^.

Multilayer growth of BeWo cells was observed, which are not contact inhibited^7^, but interestingly TEER values did not exceed literature values. Also, regardless of the multilayer growth, the underlying bioprinted “stromal compartment” seemed to have an effect on the overall conductivity and permeability of the model. For placental tissue electrical resistance is unknown, and therefore, currently TEER measurements can only be used for model comparison, but not for evaluation of physiology. For further application and to compare the presented model to other in vitro and ex vivo models, it would be insightful to test it in the future with a substance that normally crosses the placental barrier like antipyrine, caffeine or indomethacin^58,59^.

Cocultures of BeWo, fibroblast and endothelial cells were established and cultured for 12 days, during which the architecture was maintained. Arrangement of cells was visualized in immunofluorescence stainings of sections, in which nuclei in all cellular layers showed integrity and cell bodies contained typical markers, indicating successful cell culture of viable cells. Since we did not expect a profound change in conductivity nor in permeability in coculture with BeWo cells^6^, but endothelial cells may rather dedifferentiate in vitro^53^, we investigated specific endothelial cell markers. Besides confluent growth, cells showed endothelial markers found in placenta^60^.

We think the placenta inspired model to be interesting for future substance testing. Integration of primary trophoblast cells will improve the model, since BeWo cells do not reflect trophoblast physiology in every aspect, due to their malignant origin. For example, although similar asymmetric glucose transport activity occurs in both syncytiotrophoblast and BeWo cells, the glucose transporter isoform expression is different^61,62^. An alternative way to improve the model may also be to enhance fusion of BeWo cells using forskolin^32^ or galectin 3^63^.

Although choriocarcinoma cells do not reflect all characteristics of primary trophoblast cells, differences in placental structure and metabolism between human and other species suggest that model systems based on human cells may provide more relevant information in toxicology studies^64–66^. Schmidt et al. recapitulated human specific features of the placenta^12^, for example the expression of certain miRNAs. The expression profile of one human-specific miRNA cluster, C19MC, is closely matched between primary human trophoblast cells and choriocarcinoma cell lines^67,68^. Although the exact role of C19MC is not known yet, it is assumed to be altered in pregnancy pathologies, due to its placenta-specific expression^69,70^.

Furthermore, in favour of an in vitro model, the BeWo b30 clone resembles the villous trophoblast expression of multi drug resistance-related protein (*MDR-1*, P-glycoprotein) and breast cancer resistance protein (*BCRP*)^71^, but other BeWo clones do not^72,73^. Expression of both of these transporters is important in trans-trophoblast transport, and thus, in protecting the organism from toxic xenobiotics.

The placenta inspired model was cultured for 12 days, during which the integrity of the architecture persisted. It rather reflects an early placental barrier, since it includes fibroblasts and a stromal compartment, which thins during pregnancy and eventually vanishes in the term placental barrier, where endothelial and trophoblastic basement membrane fuse^24,74,75^.

The ability to test substances on an early placental model is valuable, since it corresponds to the time of organogenesis, when the demand for nutrients increases and the fetus is most susceptible to toxic influences^76^. We believe the placental inspired model we present here reflects architectural features of the early placental barrier, which may be advantageous for further development towards substance testing and could complement a testing cascade of placental models with increasing complexity^13^.

To summarize, the current work advances existing culture systems by eliminating technical filter membranes and providing a more natural environment through a novel 3D bioprinted membrane system, the Membrick, in a standard 24 well format. The biological membrane is based on extracellular matrix, thus biodegradable, transparent, allows for continuous, non-invasive microscopy and is highly permeable to solutes and molecules up to 3 kDa. Epithelial as well as endothelial cells were shown to reach confluency on the biological membrane, enabling the creation of barrier functionality while excluding any potential unspecific effect of a filter membrane.

Further, we successfully integrated human placental fibroblasts into the biological membrane, mimicking villous stromal tissue and established a coculture with three different placental cell types on the Membrick. This represents a novel complex placenta inspired model. The ability to create more complex models by integrating a stromal compartment may enable a better understanding of substance translocation, for example across an early placenta.

Moreover we suggest the biological membrane may enable the recreation of different organ barriers. Its optical characteristics and the biological membrane’s format may be interesting for automated applications, e.g. in uptake and transfer studies. Further, patient cells may be integrated for personalized medicine applications. Overall, cell culture and substance testing will gain relevance by using a filter membrane-free 3D environment.

## Methods

### Cell culture insert with biological membrane

Biological membranes in 96-well format, in 24 well format holder, were manufactured using bioink based on methacrylated gelatine (GelMA). The bioink was prepared as previously described^77,78^. Briefly, 10 wt% gelatine (porcine skin type A, Merck, Darmstadt, Germany) was dissolved in CB Buffer [0.25M; pH9] and heated to $50\;^{\circ }$C. Methacrylic anhydride (Merck, Darmstadt, Germany) [0.1 ml/$\hbox {g}_{{gelatine}}$]was added dropwise and the reaction mixture stirred for 3h to result in GelMA. The GelMA was subsequently dialysed against desalted $\hbox {H}_{{2}}$O and filtered. Afterwards the GelMA was shock frozen in $\hbox {N}_{2 liq}$ and lyophilized for storage and precise adjustment of bioink concentration. For preparation of bioinks, GelMA was reconstituted at 10 wt% in RPMI (Merck, Darmstadt, Germany) with 10% FCS, 1% P/S (Corning, Corning, NY, USA), and mixed with 0.1 wt% photoinitator lithium phenyl-2,4,6-trimethylbenzoyl phosphinate^79,80^.

Biological membranes were printed either with or without Human Villous Mesenchymal Fibroblasts (HVMF, ScienCell, Carlsbad, CA, USA). For the latter, cells were washed with DPBS and detached using a trypsin/EDTA (TE, Corning, Corning, NY, USA) solution for 5 min at 37 $^{\circ }$C, 5% $\hbox {CO}_{{2}}$ . The reaction was stopped by adding DMEM complete medium, with 10% FCS, 1% penicillin/streptomycin (P/S) (Corning, Corning, NY, USA), to the cell suspension and cells were spun down in a microcentrifuge at $300 \times g$ for 5 min at room temperature (RT). Supernatant fluid was discarded and cells were resuspended in bioink at a density of 10$^{6}$ cells/ml. For bioprinting, bioink with cells was kept at 37 $^{\circ }$C in a shaker (Eppendorf, Hamburg, Germany) for a maximum of 30 min.

Membranes were produced by light-initiated polymerization of bioinks using the Cellbricks 3D bioprinter^81,82^. The working principle of the bioprinter is the following; a motorized printing head on a z-axis holds the Membrick body. The Membrick body is the structure, into which the GelMA membrane is printed into. Located below the printing head is an ink reservoir, which holds the bioink (with or without cells). The z-axis is calibrated for the printing process to move the Membrick body into the bioink. The bottom of the ink reservoir is clear, and light is projected from a digital light projection (DLP) unit through the bottom of the reservoir, so the bioink polymerizes and forms the hydrogel membrane within the Membrick body. Membrick bodies with integrated biological membranes (Membricks) were subsequently transferred to DPBS or cultivation medium. ThinCerts in 24 well format (Corning, Corning, NY, USA) with PET membrane and $1\;\upmu$m pores were used for comparison.

### Thickness measurements

Hydrogel membranes were fabricated following the Membrick fabrication procedure, (see above,) using 10% GelMA. Membranes were cut out of the body by running a scalpel through the membrane, along the edge of the aperture. Using a scalpel or razor blade, cross sections were then cut in different secants, flipped onto their side and measured using a Biorevo BZ-9000 (Keyence, Osaka, Japan). At least three sections were measured per sample (${\text{ n }}=3$), where each section was measured at three different sites. Averages of sections were used for graphical representation.

### Mechanical testing

Hydrogel discs were printed with 6 mm diameter and 3 mm height, using the DLP based bioprinter and 10% GelMA as described above. Discs were incubated in PBS buffer for 48 hours to swell, before pictures were obtained (BZ-9000, Keyence, Osaka, Japan) and diameters were measured using the image analyzing software BZ-Analyzer II (Keyence, Osaka, Japan). Following stress-strain curves were created by cyclic compression tests (${\text{ n }}=5$) for ${\text{ n }}=3$ discs using a texture analyzer (2.5 kN RetroLine, ZwickRoell, Ulm, Germany). The compressive Young’s modulus was calculated using the diameter of discs after swelling and slopes between 0 and 10% strain.

### Permeability assays

The biological membrane presented in this paper was characterized for its permeability towards molecules of different weight: 70 kDa Dextran-FITC (DF) together with 3 kDa Dextran-Texas Red (DTR) (ThermoFisher, Waltham, MA, USA) or 457 Da Lucifer Yellow (LY) (Merck, Darmstadt, Germany), was applied onto technical or biological membrane at $20\;\upmu$g/ml in $200\;\upmu$l DMEM, phenol red free, 1% P/S and 2 mM l-glutamine (Corning, Corning, New York). Liquid volumes on the membrane and in 24 well were chosen to result in equalized levels, with $200\;\upmu$l in cell culture insert (on technical or biological membrane) and $1068\;\upmu$l or $967\;\upmu$l respectively in 24 well. To compare permeability of the two systems, the permeated amount of substance at a time point was normalized for volume and area of membrane. Volumes of $25\;\upmu$l were sampled from both liquid compartments every other hour (in DF/DTR assay), or every hour (in LY assay) and measured together with a standard dilution series of the respective molecule using a FluoStar Omega Microplate reader (BMG Labtech, Ortenberg, Germany) at emission/excitation of 584/620 for DTR and 485/520 nm for DF and LY. Sampled volumes were returned after each measurement. At every time point volumes in both compartments, on the membrane and in well, were measured using automated pipettors (Eppendorf, Eppendorf, Germany) to account for evaporation of liquid in the well plate and to calculate absolute amounts of substance. Volumes were measured by lifting individual inserts out of the well with sterile forceps, tilting it and taking up the liquid in an electronic pipettor. Afterwards the liquid was released again into the insert. In the same way liquid in the well was measured by taking the insert out, tilting the 24 well plate and taking the liquid up in an electronic pipettor. At least five biologically independent replicates for each condition and time point were tested.

For quantification of cellular barrier formation, permeability assays were performed every day for 7 days, using LY as paracellular pathway marker. Set-up was according to permeability assays, with a single sampling time point at 4 h and using Microvasculature (MV2) Growth Medium without phenol red, supplemented according to the manufacturer instructions (Promocell, Heidelberg, Germany) and with 1% P/S (Corning, Corning, NY, USA), called MV2c. Analysis was performed using MARS Data Analysis Software (BMG Labtech, Ortenberg, Germany). For direct comparison of the permeability of PET and biological membrane for different size molecules, we calculated the apparent permeability coefficient ($P_{app}$), and found it to be the most different (and informative) at 4 h. $P_{app}$ in cm/s was calculated according to Jung et al.^83^:

1 $$\begin{aligned} P_{app} = (dQ/dt)/(A \cdot C_{0}) \end{aligned}$$

with *dQ*/*dt* being the rate of molecule permeation through the membrane over time ($\upmu$mol/s); *A* being the surface area of the membrane ($\hbox {cm}^{2}$) and $C_{0}$ as the initial concentration of substance on the membrane ($\upmu$mol/ml).

### Statistical analysis

Data is represented as median and range of at least five independent biological experiments per condition with one technical replicate each. The Mann–Whitney U-test was employed to find significant differences in permeability of technical and biological membrane. A p-value of < 0.001 was considered significant (***). Statistical analysis and graphical representation were performed in Prism 7.0 (Graphpad, San Diego, CA, USA).

### Cell culture

Human choriocarcinoma cell line BeWo b30, were kindly provided by Dr. Tina Buerki-Thurnherr (Empa, St. Gallen, Switzerland) with permission from Dr. Alan Schwartz (Washington University School of Medicine, MO, USA). Human Villous Mesenchymal Fibroblasts (HVMF) and Human Placental Vascular Endothelial Cells (HPVEC) were purchased from ScienCell (Carlsbad, CA, USA). The goal was to create a coculture with trophoblast cells, fibroblasts and endothelial cells, thus all cell types were cultured in MV2c. Cells were subcultured every 2–3 days using trypsin/EDTA solution (TE, Corning, Corning, NY, USA) and cultivated at $37\;^{\circ }$C, 5% $\hbox {CO}_{{2}}$ in humidified atmosphere. HPVEC were subcultured at 70% confluency and seeded onto fibronectin-coated (FN, Corning, Corning, NY, USA) culture flasks. FN coating was performed according to manufacturer instructions: in short, T75 flasks were incubated with $20\;\upmu$g/$\hbox {cm}^{2}$ FN over night at $37\;^{\circ }$C and 5% $\hbox {CO}_{{2}}$. Liquid was removed prior to cell seeding. HPVEC were used for experiments up to p6, BeWo were used up to p36.

### Viability assay and survival ratio

The manufacturing process opens up the possibility of integrating cells into the biological membrane. To assess cell survival and viability during the manufacturing and afterwards in culture, Membricks with $10^{7}$ HVMF/ml at passage 4 were fabricated. This cell density was chosen to result in accurate detection of viability, whereas less cells were introduced in bioprints for coculture. Number of dead and total cells was assessed at day 1, 7, 14, 21 and 28 of culture for ${\text{ n }}=3$ at each time point, according to the following protocol: MV2c medium in both compartments, in the Membrick and in the 24 well, was replaced with fresh MV2c medium containing 1:2000 CellTox Green (Promega, Madison, Wi, USA) and incubated for 15 min at $37\;^{\circ }$C and 5% $\hbox {CO}_{{2}}$. Fluorescent imaging of biological membranes was performed using Biorevo BZ-9000 (Keyence, Osaka, Japan). At $10 \times$ magnification z-stack images were acquired for three different frames. Z-stacks were analyzed using BZ Analyzer II (Keyence, Osaka, Japan) by using full focus function and manual cell counting. Volume of analyzed areas was calculated by stack height and picture dimensions and averaged over the three z-stack areas of the same biological membrane. Total number of cells in the biological membrane was acquired by the following protocol: Membricks were washed with DPBS and permeation area of the biological membrane was cut out of the body using a scalpel. Membranes were then incubated in reaction tubes with $10 \times$ TE (Corning, Corning, NY, USA) solution for 1 h in a $37\;^{\circ }$C water bath with occasional agitation. Afterwards, tubes were mixed by flicking and samples taken with Via1-Cassette (ChemoMetec, Allerod, Denmark). Total cell number was analyzed using an automated cell counter (ChemoMetec, Allerod, Denmark). Cell number was normalized for dilution and volume of biological membrane and divided by number of dead cells in the respective membrane. As an example: on day 1 around $2.5 \times 10^5$ cells were counted per membrane using an automated cell counter, which reflects the target cell concentration in the bioink of $10^7$ cells/ml. In z-stacks of three different areas of the membrane about 400–500 dead cells were manually counted, each in approximately a tenth of the total membrane volume. Percentages of living cells are displayed for individual biological membranes.

### Cell culture on biological membrane

Biological membranes were incubated with cell culture medium for at least 1 h at $37\;^{\circ }$C, 5% $\hbox {CO}_{{2}}$. For (co)cultures with endothelial cells, HPVEC were seeded first on the basolateral side of the membrane: medium from the inside was removed and inserts were placed upside down onto a 12-well plate, with 1 ml PBS in each well to ensure a humid atmosphere. HPVEC were added within $50\;\upmu$l medium on top, at a density of $10^{6}$ cells/$\hbox {cm}^{2}$, making sure to cover the whole seeding area. With the lid closed, 12-well plates were incubated for 2 h at $37\;^{\circ }$C, 5% $\hbox {CO}_{{2}}$. Afterwards inserts were turned right side up, and placed hanging in 1 ml fresh medium on a 24 well plate, before $150\;\upmu$l medium were added onto the membranes. To compare monotypic HPVEC culture on biological and filter membrane, ThinCerts with PET membrane and $1\;\upmu$m pores (Greiner Bio-One, Kremsmuenster, Austria) were employed. Before equilibration with media, the PET membranes were coated with $50\;\upmu$g/$\hbox {cm}^2$ placental collagen IV (Merck, Darmstadt, Germany) for 1 h at $37\;^{\circ }$C, 5% $\hbox {CO}_{{2}}$ and washed twice with DPBS. Monotypic HPVEC cultures were performed in Endothelial Cell Medium with 5% FCS, and 1% endothelial cell growth supplement (ScienCell, Carlsbad, CA, USA) as recommended by the provider. Cell seeding followed as described above. BeWo cells were added for cocultures on biological membrane in $50\;\upmu$l MV2c at a density of $10^{6}$ cells/$\hbox {cm}^{2}$, and plates were moved in an 8-shape to distribute cells equally on the cultivation area. Microscopic pictures were taken to follow growth using a Biorevo BZ-9000 (Keyence, Osaka, Japan).

### Transepithelial electrical resistance (TEER)

Transepithelial electrical resistance (TEER) measurements indicate the conductivity of a cell layer in between two liquid compartments. The principle is based on two electrodes, on either side of the cell culture, each in one liquid compartment. A voltage is applied to both electrodes and any current that occurs in between them is measured. If both electrodes are in a single compartment, filled with a buffer, ions can move freely and thus, a current can be measured (resistance is close to zero). A confluent cell layer in between the electrodes may act as a resistor, as ions cannot pass freely anymore. In general, the more and tighter the intercellular connections are within a cell layer, the higher the resistance of the layer appear. Since the underlying scaffold displays a certain resistance by itself, this value is subtracted from the sample value and results in the intrinsic resistance of the cell layer. TEER of cell cultures on Membrick was measured every day as an indicator for cellular barrier formation. Medium was replaced each day with fresh MV2c and the Membricks were incubated at $37\;^{\circ }$C, 5% $\hbox {CO}_{{2}}$ after medium exchange for 1 h before measurements to equilibrate. TEER measurements were performed using a STX2 chopstick electrode and EVOM2 (World Precision Instruments Inc., Sarasota, USA). Additionally, we designed and 3D printed a custom holder for the electrode, ensuring reproducible placement and preventing contact with the cell layer. Cultures were kept at $37\;^{\circ }$C during TEER measurements by placing the 24 well culture plates on warming mats, normally used in animal care. Measurements were repeated thrice for each sample, every time advancing the placement of the electrode by $120^{\circ }$. Membricks, with biological membrane but without cells, were measured as intrinsic control and subtracted from sample values. Further, values were corrected for the permeation area (*0.28 $\hbox {cm}^2$) to yield normalized resistance (in ${\Omega } \hbox {cm}^2$).

### Immunofluorescence

Antibody stainings were performed to identify cell types by cell type specific marker expression. Cell cultures on Membricks were fixed in icy acetone or formalin for 5 min at RT and biological membranes were cut out of the Membrick body. Further staining of whole membranes was carried out in reaction tubes. Permeabilization followed for formalin-fixed samples in 0.2% Triton X-100 (ThermoFisher, Waltham, MA, USA) for 10 min, at RT. For cross-sectioning, biological membranes were transferred to TissueTek (Sakura, Alphen aan den Rijn, Netherlands) and incubated at $37\;^{\circ }$C for 30 min before freezing in $\hbox {N}_{2 liq}$. Cryosectioning was performed to yield sections of $8\;\upmu$m thickness, which were fixed in icy acetone for 10 min prior to staining. After blocking for 20 min in DPBS with 10% goat serum at RT, samples were incubated with 1:100 primary antibody mouse-anti-human CK7 (Agilent, Santa Clara, CA, USA) or mouse-anti-human VE-Cad (Santa Cruz Biotechnology, Dallas, TX, USA), mouse-anti-human $\beta$-catenin ($\beta$-Cat) (ThermoFisher, Waltham, MA, USA) and rabbit-anti-human vimentin, or mouse-anti-human CD31 (ThermoFisher, Waltham, MA, USA) at $4\;^{\circ }$C overnight and with fluorophore-coupled antibody sheep-anti-human von Willebrand factor (vWF)-FITC (Abcam, Burlingame, CA, USA, 1:100) and $4'$,$6'$-diamin-$2'$-phenylindol (DAPI, Merck, Darmstadt, Germany, 1:200) for 45 min at RT. Secondary antibodies goat-anti-rabbit CF488A and goat-anti-mouse CF594 (Biotium, Fremont, CA, USA) were incubated at 1:200 in DPBS, together with DAPI (Roche, Basel, Switzerland) for 45 min at RT. Samples were covered in mounting solution (VWR, Radnor, PA, USA) and imaged with Biorevo BZ-9000 (Keyence, Osaka, Japan). Control staining was performed with secondary antibodies only to exclude unspecific antibody reaction.

## Supplementary information

Supplementary Information.
