# Supplementary material for: Inspired by the human placenta: a novel 3D bioprinted membrane system to create barrier models
Source: Sci Rep. 2020 Sep 24;10:15606. doi: 10.1038/s41598-020-72559-6 (PMC7515925; doi:10.1038/s41598-020-72559-6)
Supplement: Supplementary file 1 — Supplementary Information. [file 41598_2020_72559_MOESM1_ESM.pdf]

Inspired by the human placenta - a novel 3D bioprinted membrane system to create barrier models *Supplementary Information*

Anna-Elisabeth Kreuder[1,2*], Aramis Bolaños-Rosales[1,2], Christopher Palmer[2], Alexander Thomas[1,2], Michel-Andreas Geiger[2], Tobias Lam[2], Anna-Klara Amler[1,2], Udo R. Markert[3], Roland Lauster[1], and Lutz Kloke[2*]

[1]Technical University of Berlin, Medical Biotechnology, Berlin, 13355, Germany
[2]Cellbricks GmbH, Berlin, 13355, Germany
[3]University Hospital Jena, Department of Obstetrics, Placenta Lab, 07747 Jena, Germany
[*]aek@cellbricks.com, lk@cellbricks.com

ABSTRACT

Barrier organ models need a scaffold structure to create a two compartment culture. Technical filter membranes used most often as scaffolds may impact cell behaviour and present a barrier themselves, ultimately limiting transferability of test results. In this work we present an alternative for technical filter membrane systems: a 3D bioprinted biological membrane in 24 well format. The biological membrane, based on extracellular matrix (ECM), is highly permeable and presents a natural 3D environment for cell culture. Inspired by the human placenta we established a coculture of a trophoblast-derived cell line (BeWo b30), together with primary placental fibroblasts within the biological membrane (simulating villous stroma) and primary human placental endothelial cells - representing three cellular components of the human placental villus. All cell types maintained their cell type specific marker expression after two weeks of coculture on the biological membrane. In permeability assays the trophoblast layer developed a barrier on the biological membrane, which was even more pronounced when cocultured with fibroblasts. In this work we present a filter membrane free scaffold, we characterize its properties and assess its suitability for cell culture and barrier models. Further we show a novel placenta inspired model in a complex bioprinted coculture. In the absence of an artificial filter membrane, we demonstrate barrier architecture and functionality.

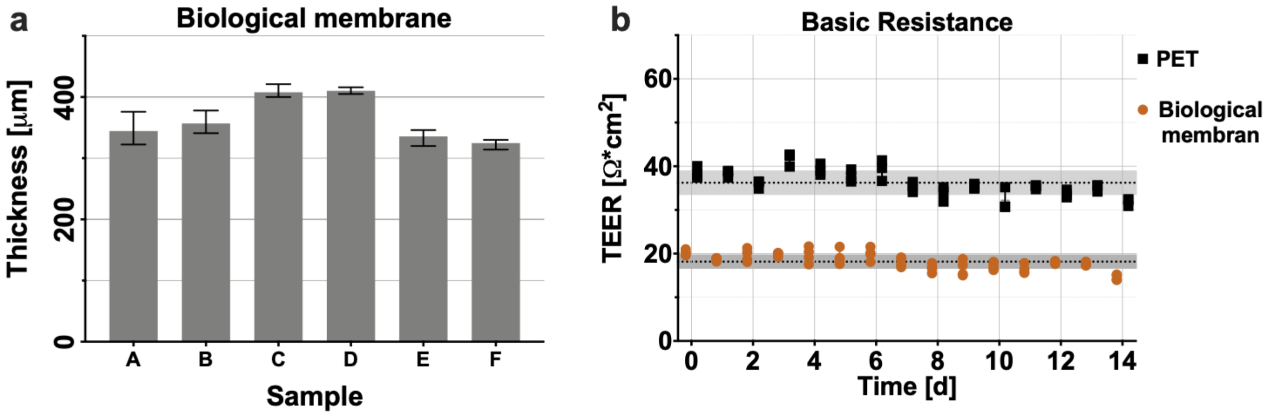

Figure 1. Characterisation of biological membrane. The bioprinted membrane is 363.3±31.5 μm (n=6) thick, average thickness of individual bioprints are displayed with standard deviation, measured at different sites (n=4) (a). Basic electrical resistance of biological membrane compared to technical membrane over two weeks (b, PET, n=3 vs. biological membrane, n=4). Values are represented as median with range. Dotted lines and grey area represent means and standard deviation, respectively.

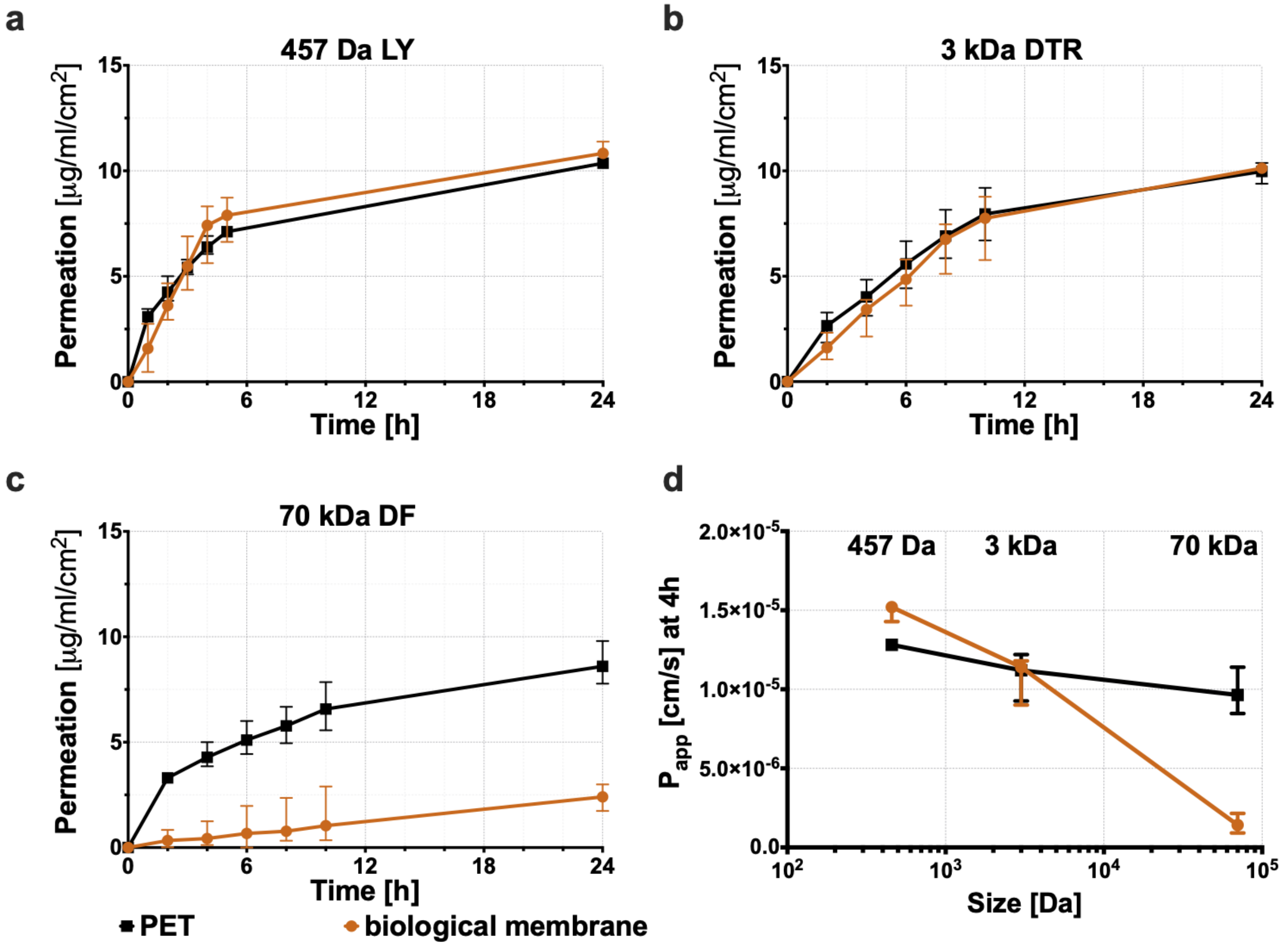

Figure 2. Permeability of biological membrane compared to PET with 1 μm pores. Permeability for 457 Da Lucifer Yellow (LY, a), 3 kDa Dextran Texas Red (DTR, b) and 70 kDa Dextran FITC (DF, c). Dextran permeability for biological membrane n=6, and LY n=5, permeability for PET n=7, and LY n=5. For direct comparison of permeability in PET and biological membrane the apparent permeability coefficient is displayed at 4h. Median and error are displayed.

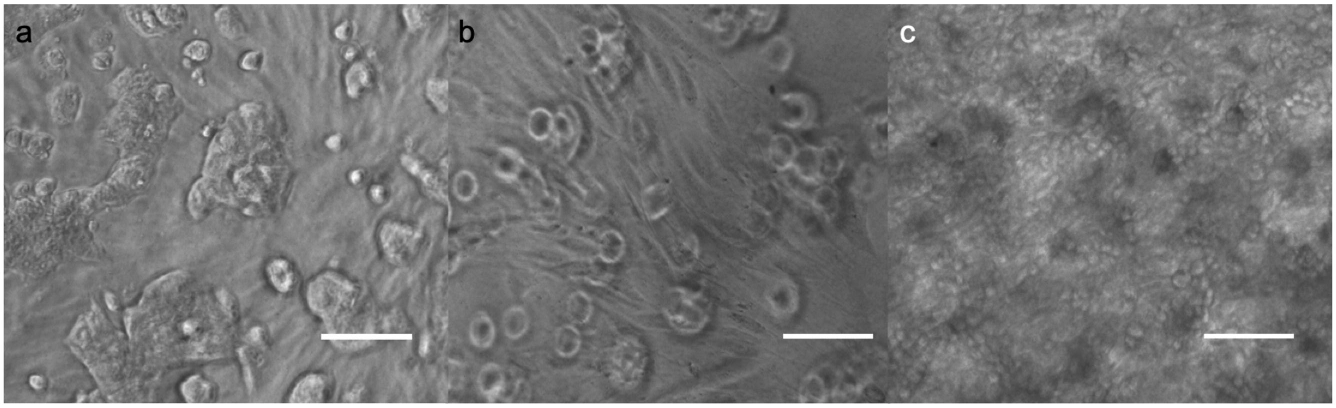

Figure 3. Visibility of apical and basolateral cell layer on the Membrick. Focus on BeWo cells (a), seeded on the apical side of the Membrick; and on HPVEC (b), seeded on the basolateral side of the Membrick. Picture frame is shifted. Basolaterally seeded HPVEC in coculture with cells in biological membrane and seeded on apical side, c. Scale bars are 100 μm

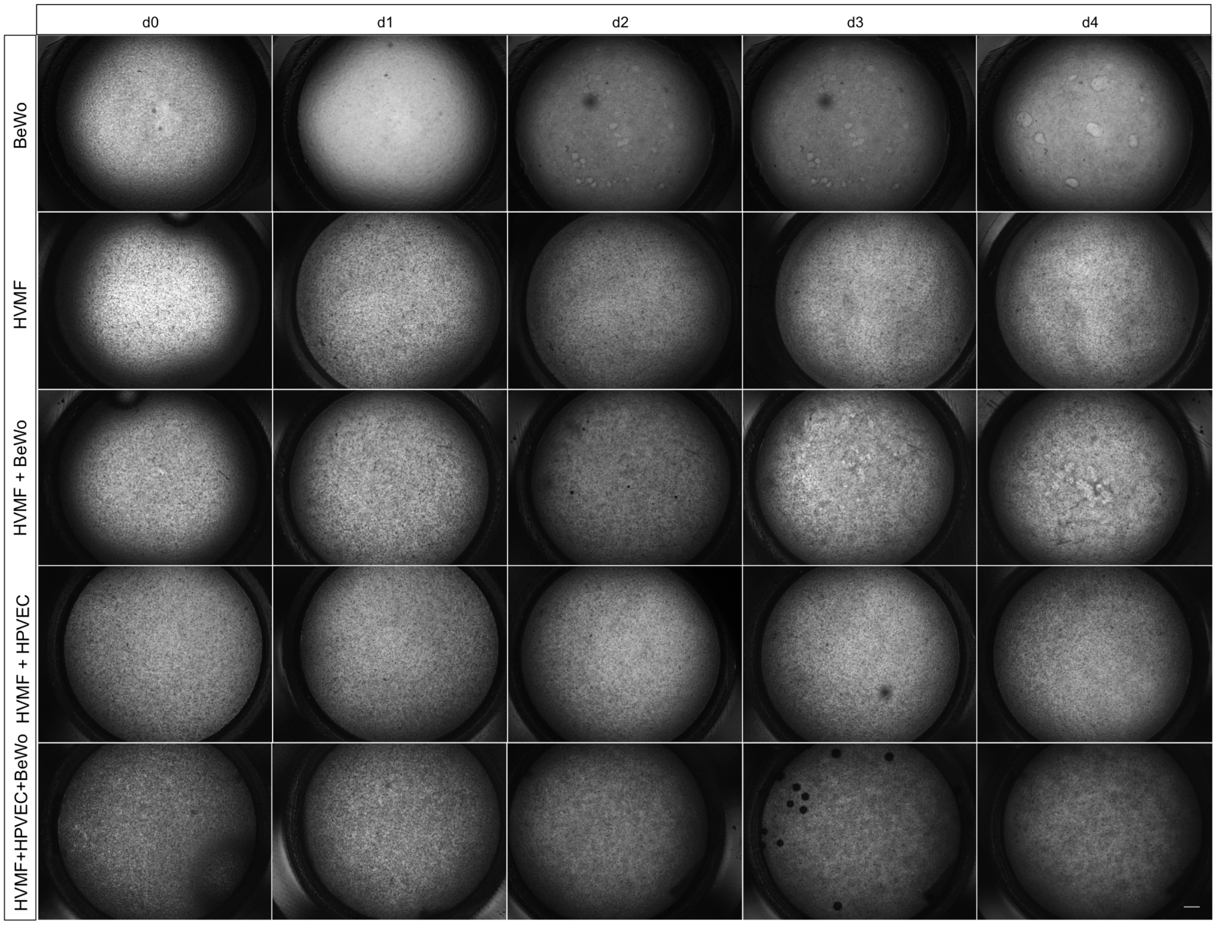

Figure 4. Timecourse of confluency in (co-) culture on the biological membrane. Trophoblast cell line (BeWo) or placental fibroblasts (HVMF) were cultivated on/in biological membranes in monotypic culture, in coculture (HVMF+BeWo) or in coculture with villous endothelial cells (HVMF+HPVEC+BeWo). The same individual samples were monitored for the entire duration of culture. Scale bar 500 μm. On day 2 and 3, a bubble in the apical media led to dark spots in first and fourth condition. In the last condition multiple small bubbles were trapped below the Membrick after medium exchange.

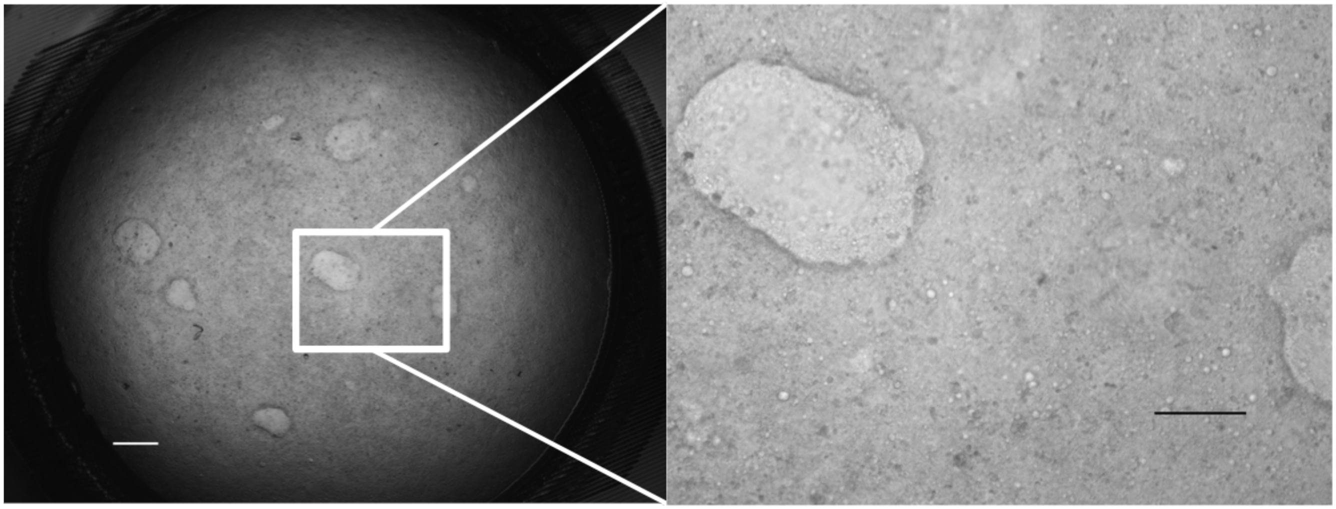

Figure 5. Details of BeWo culture on Membrick, day 4. White scale bar 500 μm black scale bar 200 μm.

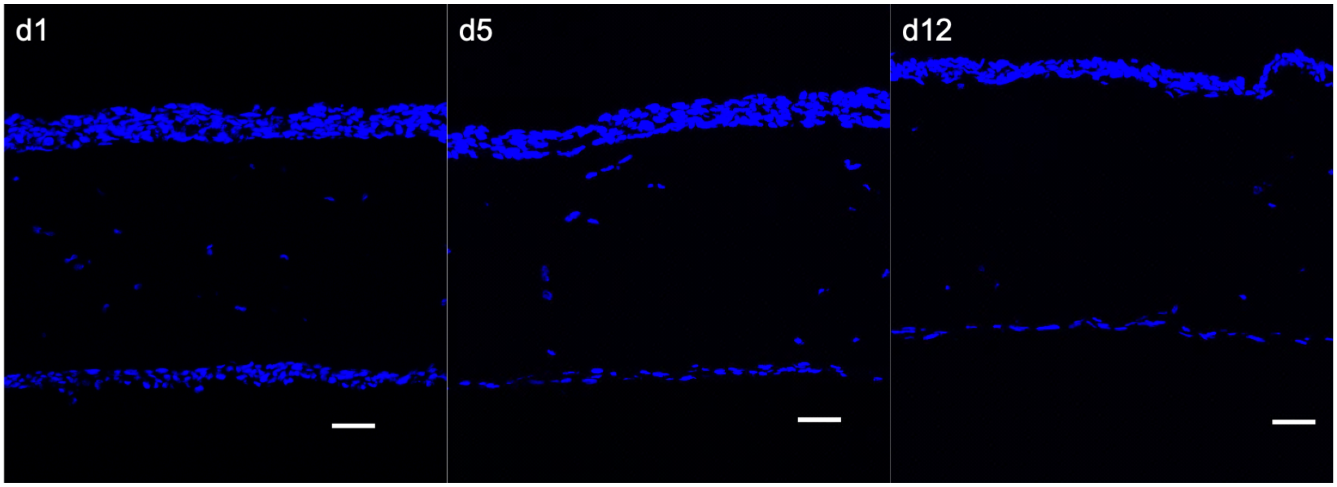

Figure 6. Thickness of biological membrane with HVMF (in GelMA) and BeWo (apical), day one coculture including HPVEC (basolateral). DAPI staining indicates cells and thickness of biological membrane on day 1, 5 and 12 of coculture.
